# Movement ecology of diploid and triploid grass carp in a large reservoir and upstream tributaries

**DOI:** 10.1371/journal.pone.0281128

**Published:** 2023-03-08

**Authors:** Tyler M. Hessler, Duane C. Chapman, Craig P. Paukert, Jeffrey C. Jolley, Michael E. Byrne

**Affiliations:** 1 U.S. Geological Survey, Columbia Environmental Research Center, Columbia, Missouri, United States of America; 2 U.S. Geological Survey, Missouri Cooperative Fish and Wildlife Research Unit, School of Natural Resources, University of Missouri, Columbia, Missouri, United States of America; 3 Michigan Department of Natural Resources, Bay City Customer Service Center, Bay City, Michigan, United States of America; 4 School of Natural Resources, University of Missouri, Columbia, Missouri, United States of America; Complutense University of Madrid: Universidad Complutense de Madrid, SPAIN

## Abstract

Grass carp *Ctenopharyngodon idella*, is an herbivorous fish originally brought to North America from Asia in 1963 to control nuisance aquatic vegetation. Since their arrival, detrimental alterations to aquatic ecosystems have sometimes occurred in waterways where they were initially stocked and into which they have escaped. The movements of grass carp from lentic systems into tributaries required for spawning is poorly understood, and understanding environmental conditions associated with upstream migrations may aid in management of the species. We stocked 43 fertile diploid and 43 sterile triploid grass carp implanted with acoustic transmitters into Truman Reservoir, Missouri, USA between January 2017 and October 2018 to characterize movements during spring and summer when spawning conditions occur. Twenty fish (11 diploid/9 triploid) exhibited upstream migration behavior in the Osage River, a major tributary, in 2018 and 2019. Migration primarily occurred in April and May, during high discharge events associated with increasing river stage when water temperatures were between 15 and 28°C. Observed migrations ranged from 3.0–108 river km in length, and six individuals were observed making multiple upstream migrations in one season. Eleven fish initiated upstream migrations while in the lentic main body of the reservoir. These findings provide some evidence for upstream migrations by diploid and triploid grass carp as well both lake and river residents. Evidence of similar upstream migration behavior by both diploid and triploid grass carp suggests that triploids may be suitable surrogates for diploids for study of movement ecology. Removal efforts in tributaries targeting periods of increasing river stage during spring may provide the best opportunity of encountering large concentrations of grass carp.

## Introduction

Originally brought to the United States in the early 1960s to control aquatic vegetation [1], the grass carp *Ctenopharyngodon idella* is an herbivorous fish native to Asia that has become widely dispersed across 45 states by means of natural dispersion and intentional stockings [2]. Grass carp can alter or significantly deplete aquatic vegetation communities in systems to which they are introduced [3–5]. As aquatic vegetation is an important source of food and shelter for native organisms [6], grass carp introductions can have detrimental effects on fauna that rely on aquatic vegetation, including birds, native fishes, and invertebrates [7]. In systems where grass carp cannot reproduce, environmental effects should persist only for the life of the fish. However, where reproduction is possible, established populations pose a persistent ecological threat if they cannot be controlled.

Within the United States, grass carp are known to reproduce naturally in large parts of the Mississippi River Basin [2], the Trinity River in Texas [8], and in the Lake Erie Basin in Ohio [9]. They often inhabit lakes and ponds, but they must broadcast their semi-buoyant eggs into flowing water [7]. The eggs are kept in suspension by turbulence, drifting for approximately one to two days depending on temperature [10]. Settlement resulting from inadequate turbulence results in high levels of egg mortality [11]. Nico et al. [12] estimated recruitment required ≥ 100 km of unimpeded river; however, FluEgg models indicate > 27% of eggs could hatch in tributaries as short as 40 km if adequate water temperatures are reached [13]. Furthermore, using 3-dimensional transport models, Heer et al. [14] indicated that successful development and hatching of some eggs could occur as little as 1 km downstream of spawning locations when accounting for eddies and areas of slower current velocity. Turbulent waters, such as at the confluence of rivers and below dams, are often used as spawning sites [15] because they provide the turbulence required for eggs to remain suspended in the water column during the water hardening process, when the eggs are denser [16]. While understanding of grass carp development and transport models indicates that grass carp might successfully reproduce and establish in reservoirs with tributaries of adequate length and hydraulic characteristics, it has rarely been documented. In North America grass carp reproduction is documented only in a few tributaries to lentic systems including Lake Erie, Truman Reservoir, Missouri, and Lake Texoma at the Oklahoma-Texas border [17–20]. We note that studies that would potentially document spawning of grass carp in reservoirs are rare, and established grass carp populations in reservoir basins may in fact be common, especially because grass carp captures are easily assumed to be escapees from pond stockings for vegetation control [21]. Persistence in lakes and reservoirs is reliant on adult fish being able to migrate in spring into tributaries suitable for successful spawning. It is well known grass carp require increased discharge and turbulence as well as water temperatures > 18°C to spawn successfully [12], but environmental conditions associated with movements of grass carp from lacustrine habitat into tributaries to spawn are not well studied. Accurate prediction of fish movement into spawning tributaries could help determine the most opportune time to remove fish in spawning aggregations.

Movement behavior in grass carp has largely been inferred from studies using sterile, triploid fish to avoid unwanted reproduction in targeted systems [22–25]. First produced in the early 1980s [26], triploids retain a third set of chromosomes, rendering triploid grass carp facultatively sterile [27]. To date, there are only three published telemetry-based studies of grass carp movement that used diploid grass carp [21, 22, 28]. Further, many states and provinces require that only triploid fish can be possessed or sold [29] and thus they are much easier to acquire by researchers [30].

An important assumption for studies that aim to use triploid grass carp as surrogates for fertile and potentially reproductive grass carp is that triploid grass carp behave similarly to fertile diploids. However, because triploid grass carp do not develop fully mature gonads [27], it is reasonable to question whether they exhibit similar spawning-related behaviors that are influenced by hormone production. Triploid female grass carp produce rudimentary gonads [31] and triploid males have only been observed to spermiate when induced artificially [32], indicating reproductive cues might be absent or diminished in triploids. No study has made direct comparisons of movement behaviors in diploid and triploid fish to determine if they behave similarly enough to assume one group behaves like the other.

Using acoustic telemetry, our objectives were to determine 1) when grass carp migrate upstream, 2) environmental conditions associated with upstream migrations, and 3) whether triploid grass carp will migrate upstream consistent with wild, diploid fish. Predicting when fish move into tributaries could assist managers in removal efforts or use of barriers to block access to spawning areas [21, 33]. We hypothesized that there would be significant differences between diploid and triploid grass carp, because triploid grass carp are sterile and would not benefit from or be inclined to make long, energetically-costly upstream migrations.

## Methods

### Study site

**Fig 1:**
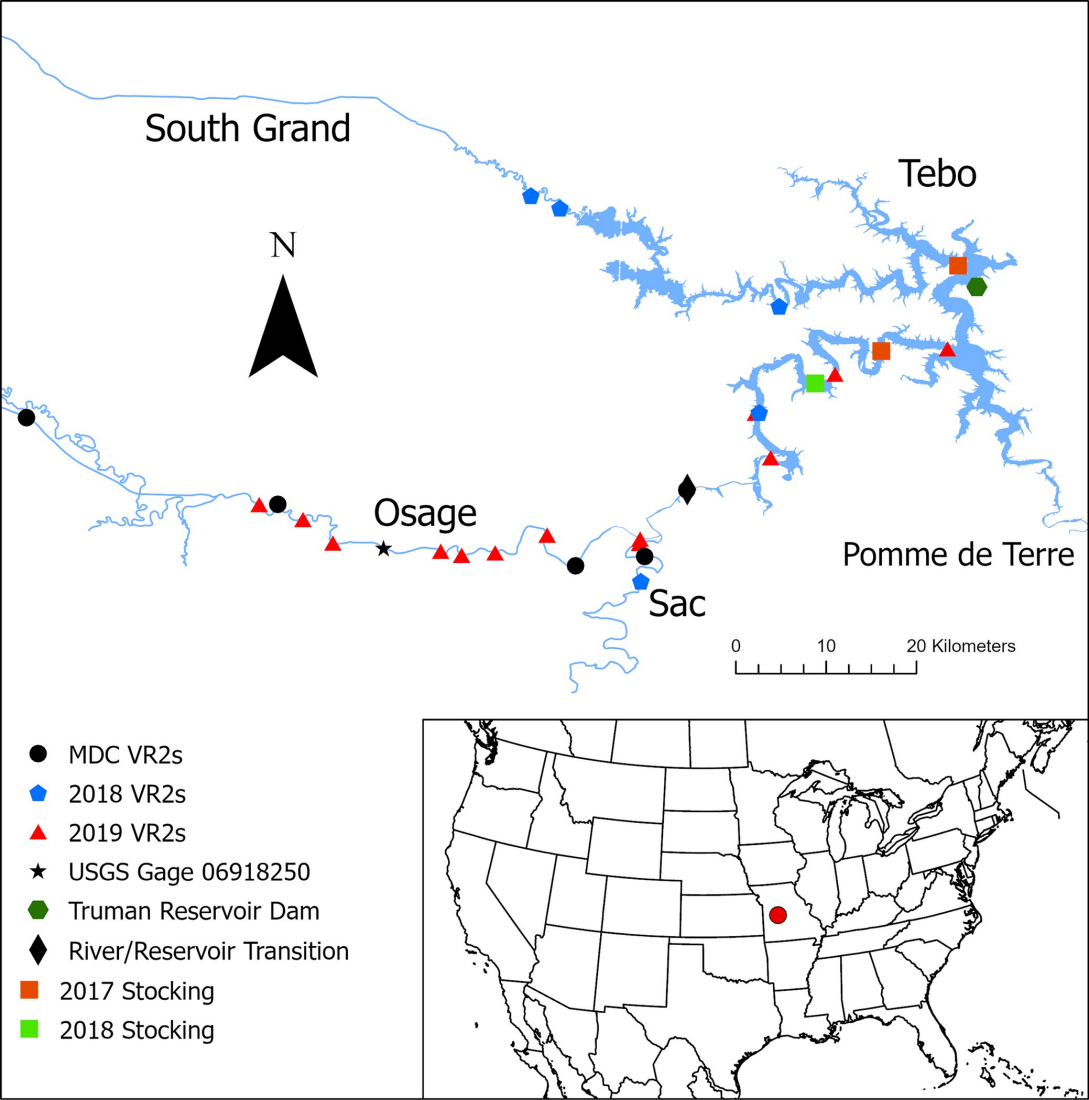
Location of tagged grass carp *Ctenopharyngodon idella* stockings and of 18 stationary acoustic receivers (VR2s) deployed while tracking them on Truman Reservoir, Missouri in 2018 and 2019. Receivers were located on the Osage River, Sac River, South Grand River, and Tebo Creek. Five Missouri Department of Conservation (MDC) receivers were in place the entirety of the study, and the other 13 were relocated from the lake proper into the tributaries from March-August.

Truman Reservoir is the largest reservoir in Missouri and was formed by the damming of the Osage River near Warsaw, Missouri in 1979 [34] by the U.S Army Corps of Engineers. Truman Reservoir has a surface area of > 22,500 ha and four major tributaries, the Osage River, Pomme de Terre River, South Grand River, and Tebo Creek (Fig 1). Another major tributary, the Sac River, empties into the Osage River just above the reservoir at river kilometer 80 (rkm, river kilometers upstream from Truman Dam). Grass carp have likely been present since the formation of the reservoir via inundation of ponds in the watershed that were stocked with grass carp for vegetation control, and potentially through natural recruitment or continued escapement from ponds in the watershed where grass carp are stocked for vegetation control [35, 36]. Because the reservoir was filled before triploid grass carp production technology was developed, and the production and stocking of diploid grass carp remains legal in Missouri [30], all escapees from ponds inundated by the initial filling of the reservoir would have been fertile, diploid fish. Many fish that later escaped from ponds in the watershed would also have been fertile. The Missouri Department of Conservation, which manages the Truman Reservoir fishery, does not stock grass carp into the reservoir [20]. With the discovery of successful spawning through the collection of fertilized eggs on four Truman Reservoir tributaries [20], it became evident that natural recruitment has likely increased the population in the system.

### Tagging and telemetry

We stocked 50 grass carp (25 diploid, 25 triploid) into Truman Reservoir on 20 January 2017 and 36 (18 diploid, 18 triploid) on 29 October 2018 [37]. The 2017 fish were divided equally between Truman State Park access (South Grand arm) and Berry Bend access (Osage arm; Fig 1). The 2018 fish were stocked at the Fox Run access (Osage arm, Fig 1). All fish were implanted with V16 acoustic transmitters (34 g, Innovasea, Bedford, Nova Scotia) in the peritoneal cavity through a 25 mm incision on the ventral side following methods descried by Schramm Jr. and Black [38]. Incisions were made halfway between the pectoral and pelvic fins, slightly to one side of the midline and were closed with 2–4 surgeon’s knots. Fish were injected with oxytetracycline to prevent infection. Tag weight never exceeded 3% of fish body weight. Transmitters had a 45 s nominal delay with an expected tag life of 1,825 days. Fish stocked in 2017 were given one month to heal in indoor tanks and fish stocked in 2018 were given ≥ 2 months to ensure survival of surgery and that incisions closed completely to reduce tag loss. Total length at stocking ranged from 570–905 mm (mean = 660 mm, SD = 68 mm) for diploids and 550–840 mm (mean = 682 mm, SD = 76 mm) for triploids. Wet weight for diploids ranged from 2.16–10.45 kg (mean = 3.51 kg, SD = 1.45 kg) and triploids 1.78–6.66 kg (mean = 3.30 kg, SD = 1.35 kg). All fish were presumed mature because they were > 500 mm total length, the minimum threshold for maturation [29]. Sex was not determined prior to stocking. Surgery protocol was approved by the Institutional Animal Care and Use Committees of the U.S. Geological Survey’s Columbia Environmental Research Center (ACU17-001) and the University of Missouri (Protocol# 9424).

We used a combination of active and passive telemetry to detect fish movements from March—July in 2018 and 2019. Tracking was limited primarily to the Osage River and its tributaries where spawning has been documented [19] and the majority of known tagged fish were located. We considered the division between the impounded portion of the reservoir and the Osage River to be at rkm 73, where a substantial change in current was observed most of the year (Fig 1). This point is at a bridge where the river width increases substantially on the downstream end. Most available habitat downstream of this point is lacustrine. During our study, the channel downstream of this point rarely had water velocities that exceeded 0.6 m/s, the minimum threshold expected for spawning [36]. From March—April we concentrated tracking activities in the main body of Truman Reservoir to monitor upstream progression of grass carp. As fish made longer movements upstream in late April-July, we focused tracking efforts in tributaries. While in the tributaries we tracked with boats moving downstream in anticipation of detecting fish as they made upstream migrations. When a tagged fish was encountered, we triangulated its position using a VH110 directional hydrophone (Innovasea, Bedford, Nova Scotia, Canada) by identifying the direction the transmitter ping emitted the most decibels. We performed this step multiple times over a 10 to 15-minute window to determine if it was stationary or moving upstream or downstream. We recorded GPS coordinates of fish locations using an onboard depth finder (Helix 10 Mega, Humminbird, Wisconsin, USA).

Tracking effort during this portion of the study spanned > 60 rkm, not including off-channel habitat and other tributaries we encountered. Because of the expansive study area, we deployed passive acoustic receivers (VR2; Innovasea, Bedford, Nova Scotia, Canada) throughout the arms of the lake we believed might be conducive to spawning based on previous research [21, 39], or which were of adequate length and turbulence for egg development. We deployed 9 passive acoustic receivers from March—August 2018 on the Osage, South Grand, and Tebo arms (Fig 1). We did not detect upstream movements on the South Grand and Tebo arms in 2018, therefore we only deployed receivers on the Osage River and its tributaries in 2019. There were no fish detected while manual tracking in the Pomme de Terre arm during the study and because of this we did not deploy receivers in this arm. We deployed 14 receivers in both the lake and tributaries from March—August 2019. Nine of these receivers were deployed ~5 rkm apart in the Osage River while four remained in the lake. We also used data from five stationary receivers (VR2) deployed by the Missouri Department of Conservation on the Osage River between rkm 73 and rkm 161 (Fig 1). Two receivers were also located on the Sac River (Fig 1). We tested detection capability with a test tag up and downstream of proposed sites, and only deployed receivers in locations where a test tag could be detected on both banks at base flow.

### Movement data

We recorded grass carp locations in rkm upstream from the dam to characterize large-scale upstream movements. We associated locations in wider parts of the reservoir or adjacent coves to the closest point to the river channel using the rkm at that point. This method assumes the shortest distance a fish could have traveled, although the distance could be longer if a fish traveled in a nonlinear path. Distance between locations was calculated as the shortest path a fish could take through the reservoir and tributaries upstream.

We cannot confirm grass carp that made large-scale upstream movements spawned or were intent on spawning, but seasonal upstream migrations to areas suitable for spawning has been observed in grass carp [39] and other invasive carp species [40]. As such, we assumed that upstream movements during the spring/summer seasons were associated with spawning activity in our analyses. Time between detections was irregular, making it difficult to statistically compare movement behavior among individual fish. Therefore, we scaled individual fish movement to regular 12-h time steps and estimated the distance moved (rkm) using the ‘redisltraj’ function in the package ‘adehabitatLT’ [41] in R [42]. We used 12-h steps because consecutive detections at different locations were rarely < 12-h and intervals greater than this may miss quick movements associated with the start of a potential migratory run. Following Acre et al. [43], we identified individual 12-h steps as part of a migration when distance moved was ≥ 85^th^ percentile of all observed distances moved over a 12-h period. Each movement step was then binomially categorized as part of a migration (1) or not (0).

We quantified river conditions based on data from the Osage River gage at rkm 116.7 (USGS gage 06918250). Temperature (°C), discharge (m^3^/s), and stage (m) are measured every 15 minutes at this location. We are aware that conditions were unlikely to be exactly homogeneous throughout the study area; however, as it was not feasible to measure the entire river, we make the necessary simplifying assumption that measurements at this location were representative of general conditions of discharge and temperature. River conditions along movement paths were based on gauge readings at the time of each 12-h interpolated location. The importance of these variables for successful spawning of grass carp is well documented, including ideal temperatures for optimal ripening and development of eggs post fertilization as well as higher velocities and turbulence associated with increased discharge and river stage to keep eggs suspended prior to hatching [36, 44]. In addition, these variables have been associated with grass carp spawning movements in previous studies [45, 46]. Although other environmental variables may play a role in grass carp movements (i.e., turbidity, food availability), it was not feasible to quantify them on a large spatial scale.

### Analysis

We used a generalized additive model (GAM) with a Poisson link function to model the proportion of individuals in the telemetered population migrating upstream each day during the spawning season, and included the total number of fish with tracking data available each day as an offset term. The model was fit using the ‘gam’ function in the ‘mgcv’ package [47] in R [42]. We included daily covariates for temperature, stage, discharge, and an interaction term between stage and discharge because there was not always a linear relationship between the two in 2019 as the reservoir filled and backflow was observed further upstream. Daily covariate values represent the mean stream gauge reading for each day, and all covariates were scaled and centered. Given the long monitoring period encompassed a wide range of water temperatures through spring and summer (April–August) and considering grass carp spawning likely occurs within a relatively narrow temperature range (18–24°C; Jones et al. 2017), water temperature was included in the GAM as a smooth term to account for the nonlinear effect we anticipated temperature to have on fish movement. We modeled linear relationships between migration and all other covariates to avoid overfitting and because we had no a priori assumptions of non-linearity. Parametric coefficients and smooth terms with P values < 0.05 were considered significant. Additionally, we used Lin’s concordance correlation coefficient (CCC) to assess the fit of the predicted proportion from the GAM to the observed portion of fish migrating [48].

We used a logistic regression model to determine if ploidy (categorical), total length at stocking, and wet weight at stocking affected the probability of a fish engaging in migratory behavior. For this analysis we incorporated fish with tracking data during the spawning season (April–August). Fish were considered migratory if they were observed making ≥ 1 upstream migratory movement during this time. We scaled and centered total length and wet weight. We considered covariates significant if the 95% confidence interval of the parameter estimate did not encompass 0.

## Results

Seventeen (20%; 8 diploid and 9 triploid) of the 86 tagged fish were lost either to dropped tags or mortality and 9 (10%; 4 diploid and 5 triploid) were never located during the study. Of the remaining 60 fish, 20 (11 diploid and 9 triploid; Table 1) were detected making upstream movements [49]. We did not detect upstream movements in 2017 from the fish stocked that year. In 2018, two diploid fish of the seven (29%) individuals known to be alive and with recent detections prior to the start of the spawning season made migrations upstream into the Osage River. One of these individuals migrated ≥ 41 rkm upstream between 20 May 2018 and 21 May 2018 while the other individual migrated ≥ 50 rkm from 15 May 2018–21 May 2018. The second individual was also detected at Sac River rkm 13 on 20 May 2018, indicating that the fish traveled ≥ 56 rkm in approximately 24 h between the two rivers. Movements by these two fish coincided with a flood event (20 May 2018–22 May 2018) where the Osage River rose > 2.2 m and discharge increased from near-median flow to > 90^th^ percentile. Temperature data were not available for the river gage during this period, but water temperature where one of the two individuals was actively located (rkm 109.7) on 21 May 2018 was 20.9°C. Three fish (2 diploid and 1 triploid) remained in the lake throughout the spawning season and one triploid remained in the Sac River, a major tributary to the Osage River, but was not detected making upstream movements. It is possible one diploid migrated into Tebo Creek in 2018, but only one passive receiver was deployed, and directionality could not be determined. No fish were detected by receivers on the South Grand River in 2018.

**Table 1:** Upstream migrations for 20 tagged grass carp *Ctenopharyngodon idella* in Truman Reservoir, Missouri in 2019. Total lengths in millimeters (mm) and weights in kilograms (kg) were at time of tag implantation. Distance traveled measured in river kilometers (rkm) upstream from Truman Reservoir Dam.

Fish ID	Ploidy	Total Length (mm)	Weight (kg)	Start Location (rkm)	End Location (rkm)	Total Distance Traveled (rkm)
9235	Triploid	740	4.83	82.4	127.1	44.7
9236	Triploid	800	5.81	82.4	136.1	53.7
9237	Triploid	785	4.67	53.5	62.7	9.2
9240	Triploid	745	4.98	73.1	96.0	22.9
9246	Triploid	750	4.51	13.4	121.5	108.1
9247	Diploid	712	4.19	82.2	121.5	39.3
9247	Diploid	712	4.19	107.6	121.5	13.9
9247	Diploid	712	4.19	82.4	111.4	29.0
9252	Diploid	905	10.45	24.7	28.1	3.4
9254	Diploid	608	2.76	37.5	53.1	15.6
9255	Diploid	810	6.91	13.4	53.1	39.7
9256	Diploid	685	4.47	82.4	96.0	13.6
9257	Diploid	790	5.99	52.8	121.1	68.3
9257	Diploid	790	5.99	89.8	135.6	45.8
9259	Triploid	758	4.78	13.4	82.2	68.8
9259	Triploid	758	4.78	82.2	111.4	29.2
9260	Triploid	570	2.31	82.5	87.1	4.6
9261	Triploid	750	4.67	28.5	60.9	32.4
9261	Triploid	750	4.67	82.6	87.1	4.5
9263	Triploid	665	3.4	81.9	84.9	3.0
52502	Diploid	660	3.06	34.7	53.1	18.4
52502	Diploid	660	3.06	13.4	87.1	73.7
52506	Diploid	670	3.42	96.0	134.1	38.1
52522	Diploid	660	2.96	13.4	39.8	53.2
52523	Diploid	623	2.7	13.4	37.4	24
52524	Diploid	675	3.38	53.3	136.5	83.2
52524	Diploid	675	3.38	72.5	121.5	49.0

Record breaking flood events occurred across Truman Reservoir and its tributaries in May-June 2019. At its highest the reservoir rose > 10 m above normal pool, inundating much of the surrounding land. In 2019, 42 fish were detected and alive prior to the onset of observed migrations, but only 27 were observed with > 1 detection after 01 April (S1 Fig). Twenty (11 diploid and 9 triploid, 74%) of the 27 fish with detection data during the spawning season migrated upstream in 2019 (Table 1). Mean total length and weight at stocking for migrating fish ranged from 570–905 mm (mean = 718 mm) and 2.31–10.45 kg (mean = 4.51 kg), respectively (Table 1). Four grass carp that made upstream movements in spring-summer 2019 were stocked in January 2017, 2 of which were also observed migrating in 2018, and the remaining 16 were stocked in October 2018. Of fish that did not migrate into the Osage River, 7 (5 diploid and 2 triploid) were detected by receivers in the Osage arm of the reservoir during the spawning season. We focused resources on the Osage River in 2019, and it is possible that fish which did not migrate up the Osage River or resided in the Osage arm of the reservoir may have moved to or migrated up unmonitored tributaries of the reservoir during the spawning season. Three triploid fish that migrated up the Osage River were also detected in the Sac River, a tributary of the Osage, during the period migrations were observed. In 2019, the furthest upstream stationary receiver on the Sac River was 6.5 rkm from its confluence with the Osage River (Sac River unimpeded length = 40 rkm), making it difficult to determine if these fish made further upstream movements on the Sac River as well. As such, these detections on the Sac River were not included in model development. Due to a limited number of detections and sparse gage data in 2018, we only included the 2019 spawning season in our analysis.

**Fig 2:**
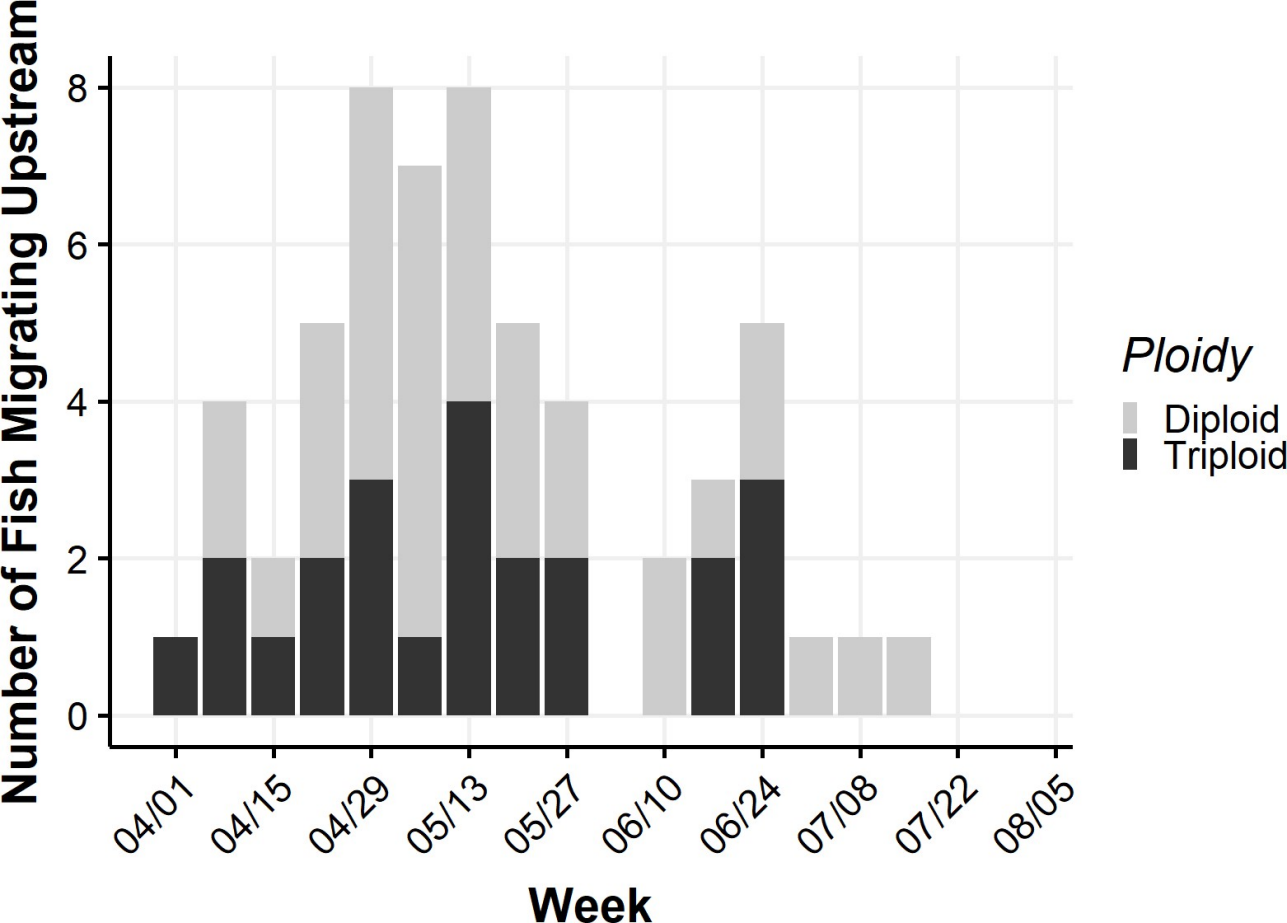
Timing of upstream migrations for 11 diploid and 9 triploid grass carp *Ctenopharyngodon idella* tracked via acoustic telemetry in the Osage River above Truman Reservoir, Missouri, 2019. Fish were included as engaging in upstream migration in any week in which ≥ 12 h tracking period indicated migratory behavior.

**Fig 3:**
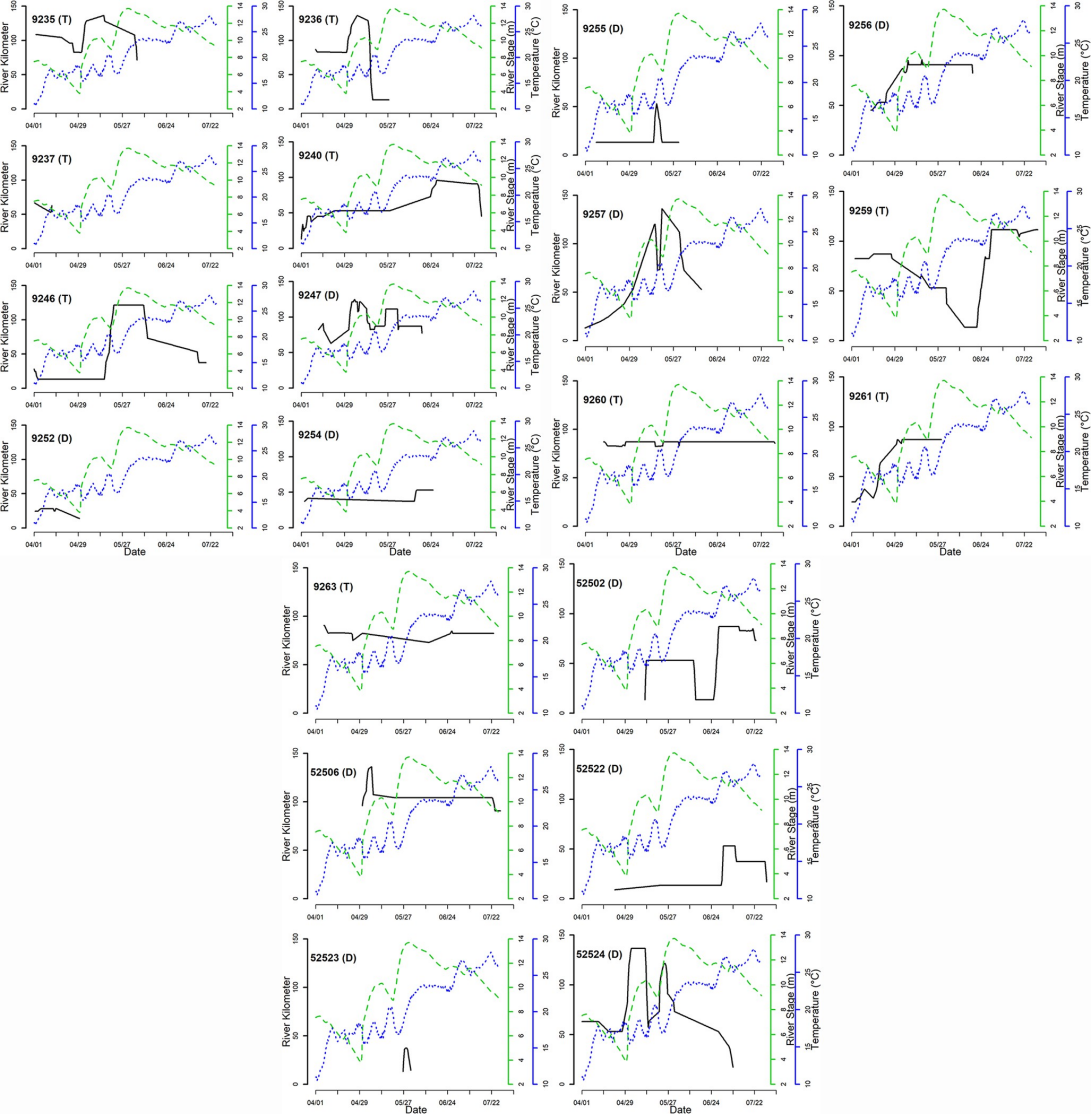
Movements of 20 tagged grass carp *Ctenopharyngodon idella* detected making upstream movements during the 2019 spawning season on the Osage River above Truman Reservoir, Missouri. The left y axis indicates distance in river kilometers (rkm) from the Truman Reservoir dam. The right y axes indicate river stage (m) and water temperature (°C) on the Osage River at Taberville, MO (USGS gage 06918250). A lack of movement for fish around the confluence of the Sac River with the Osage River (rkm 80) may be an artefact of movement between the two rivers. Numbers in the top left of each plot indicate the fish’s unique tag ID and a “D” indicates diploids and “T” triploids, more information on individuals can be found in Table 1.

Upstream migrations in 2019 occurred between 07 April and 20 July, with a peak in migratory activity occurring from late April to early May (Fig 2). There was no clear difference in migration timing between diploid and triploid grass carp (Fig 2). Eleven fish initiated upstream migrations in the lacustrine portions of the reservoir and nine began migrating above rkm 73 in the river (Fig 3). Migrations began as far downstream as rkm 13 and as far upstream as rkm 107.6 (Table 1). Fourteen fish (7 diploid and 7 triploid) made one upstream migration, five fish (3 diploid and 2 triploid) made two migrations, and one diploid made three migrations (Table 1). Of the fish that made multiple migrations, all but one returned downstream (distance = 13.9–64 rkm) before their next migration. The one fish that did not return downstream between upstream movements was a triploid that may have been resting during its upstream migration because there was only a three-day lapse between events (fish 9259, Fig 3). Total distance traveled during migrations ranged from 3.0 to 108.1 rkm (Table 1).

### Model results

**Fig 4:**
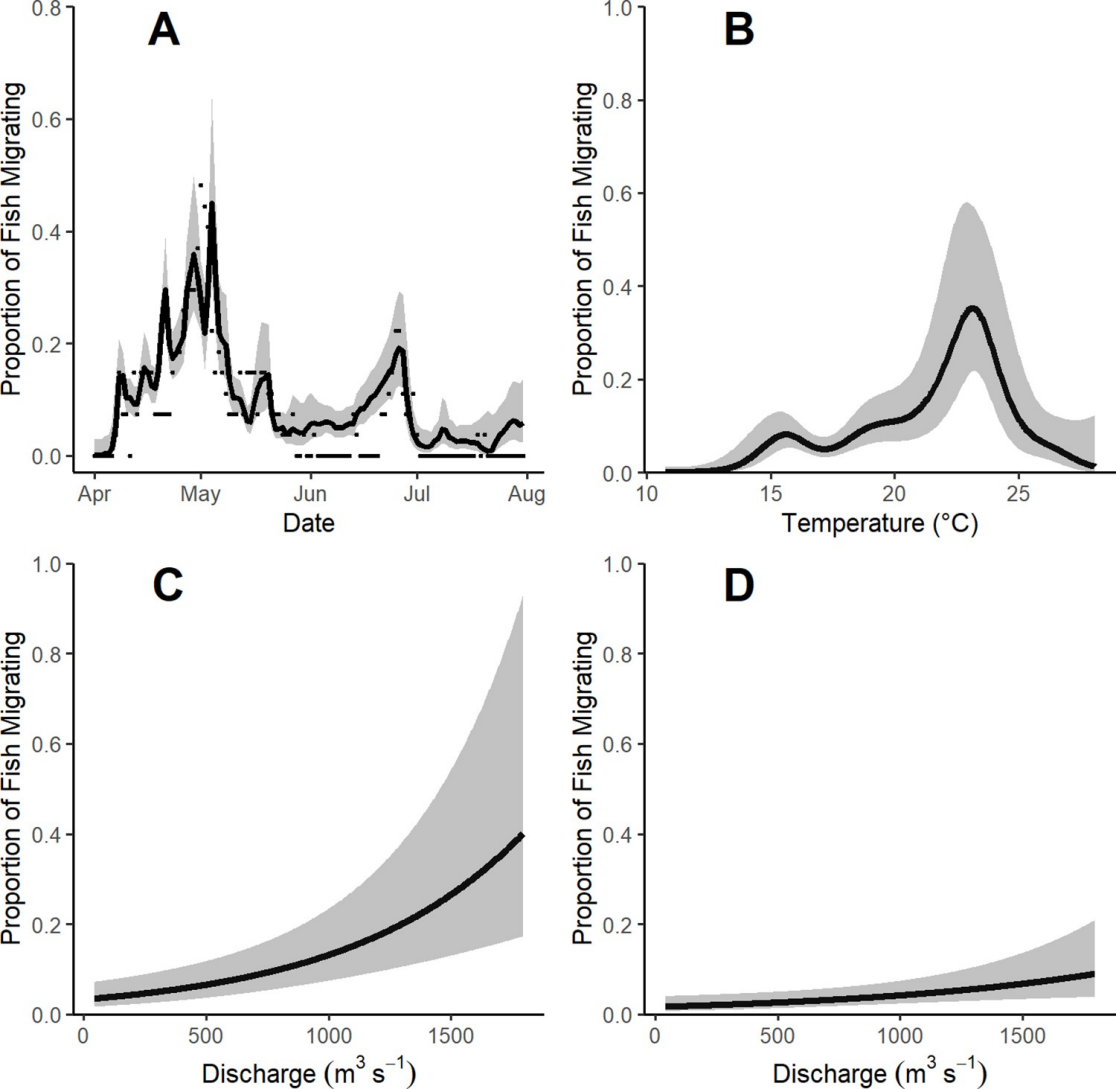
Plot A depicts the generalized additive model (GAM) predicted proportion of tagged grass carp *Ctenopharyngodon idella* migrating (black lines) with 95% confidence intervals (gray polygon) compared to the observed proportion each day (dots). Plots B through D depict the predicted proportion of tagged grass carp migrating based on a generalized additive model coefficient estimates of the GAM with 95% confidence intervals (gray polygons). Plot B indicates the predicted proportion of fish migrating upstream as a function of temperature while discharge and river stage are held constant at their mean. Plots C and D depict the predicted proportion of fish migrating upstream as a function of discharge with temperature held constant at its mean and river stage held at 11 m and 13 m, respectively.

Migratory movements in 2019 occurred in river temperatures ranging from 15.1 to 27.8°C. Most migration movements (54%) cooccurred in time with river rise events, with a mean stage increase of 0.1 m/12 h (range: -0.2–2.6 m/12 h) and mean discharge increase of 24 m^3^/s/12 h (range: -193–430 m^3^/s/12 h; Fig 3). Our GAM model included 27 migration events from 20 unique fish observed moving upstream in 2019 and an additional 7 fish that were not observed making upstream movements were also included. The model fit the data well, with a CCC value of 0.75 (Fig 4A). All parameters were informative (Table 2). Model results suggest the highest probability of fish engaging in upstream migration occurred between 16–24°C, although there was large variation (Fig 4B). The interaction between discharge and stage indicated there was a positive effect of discharge on migration; however, the strength of this effect declined as stage increased (Fig 4C and 4D).

**Table 2:** Estimates and 95% confidence intervals for parametric coefficients and estimated degrees of freedom (edf) for smooth terms for a generalized additive model (GAM) of grass carp *Ctenopharyngodon idella* spawning migration behavior on the Osage River above Truman Reservoir, Missouri, 2019.

Parametric coefficients:	Estimate	Lower 95%	Upper 95%	p-value
Intercept	-2.61	-2.90	-2.32	<0.001
Discharge	0.72	0.46	0.98	<0.001
Stage	-1.36	-1.73	-1.00	<0.001
Discharge * Stage	-0.26	-0.42	-0.11	<0.001
Smooth terms:	edf			p-value
s(Temperature)	6.75			<0.001

Twenty-seven fish were included in our logistic regression model, 20 that made migrations (11 diploid/9 triploid) and 7 that did not (4 diploid/3 triploid). None of the parameters were informative (Table 3), indicating that neither ploidy nor size had a significant effect on the probability of a fish engaging in migratory behavior.

**Table 3:** Beta coefficient estimates and 95% confidence intervals for fixed effects for a logistic regression model of grass carp *Ctenopharyngodon idella* spawning migration behavior on the Osage River above Truman Reservoir, Missouri, 2019.

Covariate	β	Lower 95%	Upper 95%
Intercept	1.92	0.26	4.39
Ploidy	0.01	-2.25	2.32
Length	-2.50	-7.03	1.22
Weight	5.02	-0.26	12.78

## Discussion

The behaviors we observed from 20 tagged grass carp in the Osage River upstream from Truman Reservoir in 2018 and 2019 suggest that water temperature and discharge were key predictors associated with upstream migrations. River conditions observed during these migrations were consistent with conditions present when Hayer et al. [20] determined grass carp to be spawning from evidence of increased eDNA concentrations and collection of fertilized eggs above Truman Reservoir [20]. Only 29% of the fish actively tracked during the spawning season in 2018 while 74% of the fish in 2019 tracked were observed making upstream migrations in the Osage River. Due to record flooding in 2019, the fish that did not migrate or were not detected after 01 April may have dispersed into backwaters and connected waterways resulting in diminished detection probability. We detected grass carp using the lower Sac River before, during, and after spawning migrations in 2019. More tracking effort on the Sac River in future work would help to determine how it is used during the spawning season. Unlike the Osage River, spawning has not been detected on the Sac River, possibly because it is regulated by an upstream dam which results in an anthropogenically altered hydrograph including abrupt decreases in discharge during the spawning season [20].

The longest upstream migration began at rkm 13.4 before ending near rkm 121.5, an example of a lake resident fish migrating 60 km through the impounded portion of the reservoir. There was no clear relationship between location and timing of a fish’s first upstream movement; in 2019 the earliest detected upstream movement was by a triploid fish residing near rkm 32.6 on 07 April 2019. The next migration detected was initiated at rkm 83.8 and did not occur until 12 April 2019. This may suggest fish were at different stages of spawning readiness and thus began migrations at different times, or fish were initiating upstream migrations for purposes other than spawning. For example, rising river stage and higher discharges could have delivered chemical and physical cues associated with flooded vegetation to trigger foraging-related upstream exploration, which may explain why five fish made a second or third upstream movement beginning downstream from the end of their last migration. Grass carp have been reported to be capable of spawning twice in one year, but it is rare [45]. Multiple discharge pulses could have delivered multiple cues, resulting in multiple migrations, even though it is likely they would have only spawned once. Direct observations of spawning behavior by tagged individuals combined with precise measures of physical processes, and perhaps, hormone profiles would be needed to improve inferences of why upstream migrations were made.

Upstream movement in the Osage River correlated with water temperature and high discharge associated with rising river stage. Although not included in our models, the upstream migrations of two fish in 2018 also coincided with optimal water temperature and high discharge. Our model suggests the greatest probability of upstream migration in 2019 coincided with temperatures ~ 16–24°C during periods of high discharge, which was associated with rising river stages except when the gaging stage was in backwater from the reservoir, during which high stages occur at low discharges (Fig 3). This large range in temperatures likely stemmed from extrapolating temperature readings from a single location to fish movements across many kilometers of river and reservoir and changes in temperature as the season progressed. It is worth noting that the significance of an interaction between discharge and stage seen in a year of historically high reservoir elevation with frequent backwater events may not be detected in more normal flow years where a more linear relationship between discharge and stage might be present. Both fish in the impounded reservoir and the river itself migrated upstream during these events, and it could be the rise in reservoir elevation that caused lake resident fish to move, like movements to spawning areas observed in centrarchids [50].

Grass carp observed migrating upstream were generally larger at stocking than average, with the mean length 47 mm longer than the mean of all tagged fish and mean weight 1.11 kg heavier than the total mean. However, length and weight were noninformative in our logistic regression model and we observed both small (length = 570 mm, weight = 2.31 kg) and large (length = 905 mm, weight = 10.45 kg) fish migrating. Additionally, the effect of ploidy in our model was almost 0 and was uninformative. An almost equal ratio of fish (11 diploid to 9 triploid) was observed migrating, with overlap between the two groups in migration timing. This is a small sample size, but the behaviors we observed indicate there is at least some inclination for triploid fish to migrate upstream during flood pulse events, a phenomenon commonly seen in their diploid counterparts.

Our model predicted increased discharge plays a role in determining when migrations occur, and that relationship is weaker as river stage increases (Fig 4). Sixteen of the 20 fish (80%) migrated upstream prior to the 14 May 2019 peak of 10.4 m and only 6 (30%) of the fish migrated during the following flood pulse when the river reached its maximum observed stage of 13.7 m on 30 May 2019. It is possible the fish that only migrated once during the initial flood event were able to successfully spawn and thus any further, larger flood events were unnecessary for those individuals. Another explanation for the lower number of migrations during the larger flood events may be diminished detectability by stationary receivers, because fish may have been able to travel in areas outside the existing river channel and thus pass receivers without detection. Extraordinary flood conditions in 2019 may be the cause of these observations and similar work in years characterized by more representative discharge regimes would help to better understand the relationship between river conditions and upstream migrations.

Upstream movements began at 15°C, which is consistent with previous observations that movements to spawning sites will begin at cooler temperatures [29] in preparation to be at sites when the 18°C threshold is reached [16, 36]. More fish were predicted to migrate upstream with optimal temperatures for spawning (16–24°C) than outside this threshold, and temperature measured during 2018 migrations also fell within this range (~ 21°C). This is further indication that these movements were likely spawning related. However, we were not able to directly confirm movements were related to spawning, but they coincided with conditions in which spawning has been inferred. Spawning in rivers has been inferred to coincide with increased discharge and river stage [19, 20, 51], which is likely advantageous for survival of drifting eggs and larvae because increased water turbulence more effectively keeps eggs distributed in the water column while drifting downstream and the increased turbulence and turbidity likely interferes with predation on the drifting eggs and larvae [16, 52]. A minimum water velocity of 0.6 or 0.7 m/s is the estimated requirement for maintaining grass carp eggs in suspension [36, 53], but maintaining eggs in suspension is dependent on turbulence, not velocity, thus lower velocities may be adequate under some conditions [54].

The results from this study indicate that fish initiate migrations upstream at different times into tributaries. Asynchronous movements by fish residing in different areas of the lake indicate that fish are entering tributaries at different times during potential spawning events. Migration events may be spread across multiple days, if not weeks. Results from this study and Harris et al. [39] in Lake Erie tributaries indicate grass carp may arrive to spawning sites over multiple peak discharge events, and it may be best to concentrate capture efforts over multiple events and before and after peaks when fish are most likely moving to and from spawning sites. Targeting fish capture in periods when they are moving to spawn and when they are congregated during the spawn may improve harvest cost-effectiveness.

In this study, both diploid and triploid grass carp made upstream migrations during favorable spawning conditions, contradictory to our initial predictions. Twelve individuals including both diploids and triploids initiated upstream migrations of similar upstream distances during the first significant water rise when conditions for spawning were favorable in 2019, which lasted from 27 April 2019–05 May 2019. Therefore, we conclude that diploid and triploid fish behave similarly in making upstream migrations into lake tributaries potentially related to spawning or for other exploratory purposes, including finding food. We do not know why triploid and diploid grass carp behaved similarly in this regard, whether the movements of triploids are motivated by physiology, schooling with diploid conspecifics, or some other factor, but these data suggest that triploid fish are suitable sterile surrogates for the study of grass carp movement ecology in lakes and their tributaries.

## Supporting information

S1 Fig Detections of 27 grass carp *Ctenopharyngodon idella* tagged with acoustic transmitters from April-August 2019 in Truman Reservoir, Missouri. The y-axis indicates the fish’s position relative to distance in river kilometers (rkm) upstream of Truman Dam. “D” indicates a diploid fish and “T” a triploid.
(TIF)
