# Supplementary figures and images for: Movement ecology of diploid and triploid grass carp in a large reservoir and upstream tributaries
Source: PLoS One. 2023 Mar 8;18(3):e0281128. doi: 10.1371/journal.pone.0281128 (PMC9994706; doi:10.1371/journal.pone.0281128)

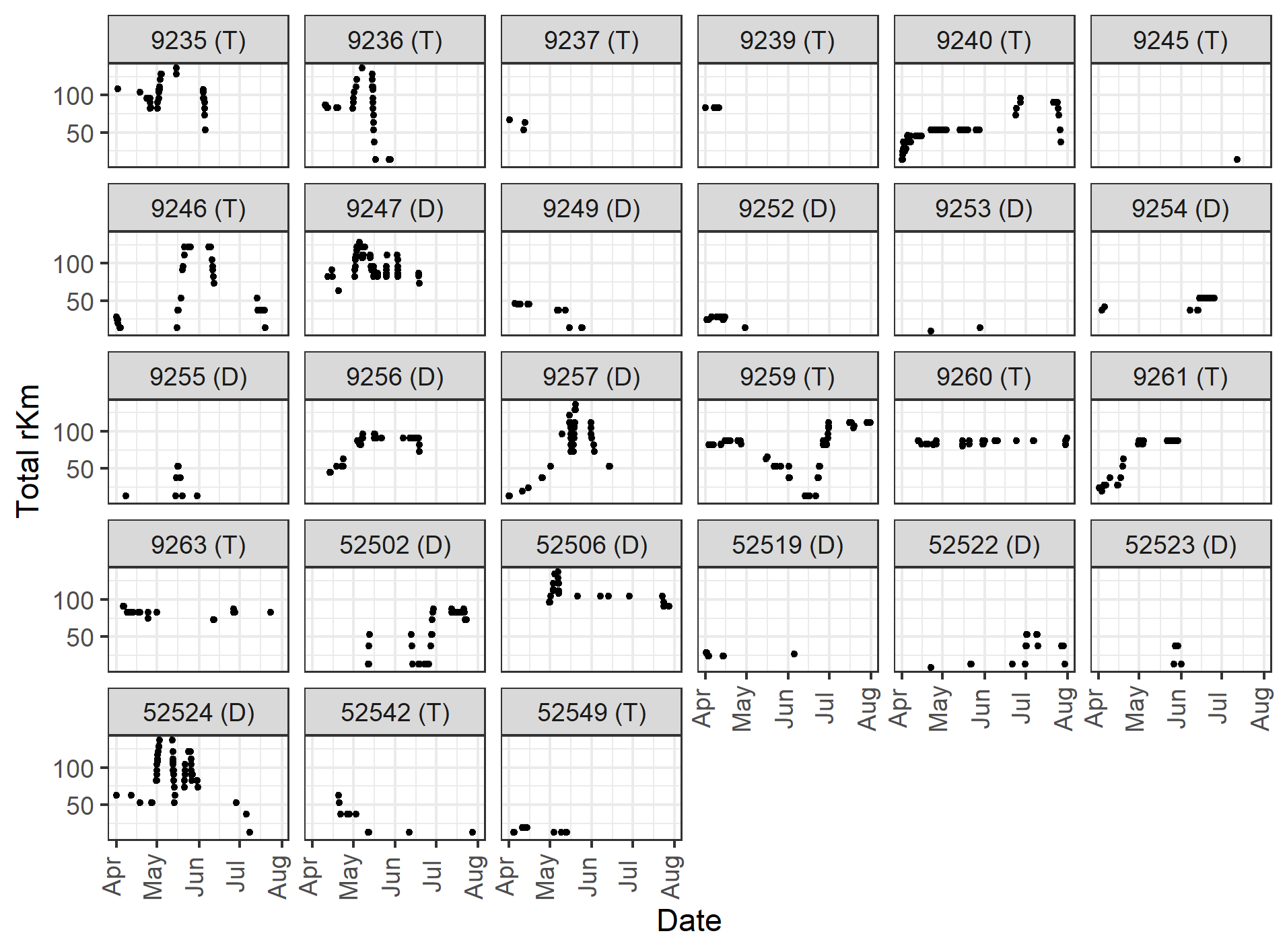

Supplement: S1 Fig — The y-axis indicates the fish’s position relative to distance in river kilometers (rkm) upstream of Truman Dam. “D” indicates a diploid fish and “T” a triploid. (TIF) [file pone.0281128.s001.tif]
